# Nano-Resveratrol: A Promising Candidate for the Treatment of Renal Toxicity Induced by Doxorubicin in Rats Through Modulation of Beclin-1 and mTOR

**DOI:** 10.3389/fphar.2022.826908

**Published:** 2022-02-25

**Authors:** Ahlam M. Alhusaini, Laila M. Fadda, Abeer M. Alanazi, Wedad S. Sarawi, Hatun A. Alomar, Hanaa M. Ali, Iman H. Hasan, Rehab Ahmed Ali

**Affiliations:** ^1^ Department of Pharmacology and Toxicology, College of Pharmacy, King Saud University, Riyadh, Saudi Arabia; ^2^ Genetics and Cytology Department, National Research Centre, Cairo, Egypt

**Keywords:** doxorubicin, nano-resveratrol, renal damage, mTOR, beclin-1

## Abstract

**Background:** Although doxorubicin (DXR) is one of the most used anticancer drugs, it can cause life-threatening renal damage. There has been no effective treatment for DXR-induced renal damage until now.

**Aim:** This work aims at examining the potential impact of nano-resveratrol (N-Resv), native resveratrol (Resv), and their combination with carvedilol (Card) against DXR-induced renal toxicity in rats and to investigate the mechanisms through which these antioxidants act to ameliorate DXR nephrotoxicity. Method: DXR was administered to rats (2 mg/kg, i.p.) twice weekly over 5 weeks. The antioxidants in question were taken 1 week before the DXR dose for 6 weeks.

**Results:** DXR exhibited an elevation in serum urea, creatinine, renal lipid peroxide levels, endoglin expression, kidney injury molecule-1 (KIM-1), and beclin-1. On the other hand, renal podocin and mTOR expression and GSH levels were declined. In addition, DNA fragmentation was markedly increased in the DXR-administered group. Treatment with either Resv or N-Resv alone or in combination with Card ameliorated the previously measured parameters.

**Conclusion:** N-Resv showed superior effectiveness relative to Resv in most of the measured parameters. Histopathological examination revealed amelioration of renal structural and cellular changes after DXR by Card and N-Resv, thus validating the previous biochemical and molecular results.

## 1 Introduction

Doxorubicin (DXR) is widely used as an efficient chemotherapeutic agent for different cancers (Minotti et al., 2004; An et al., 2017). Although DXR is a potent antineoplastic agent, its use is firmly limited due to dose-related heart, liver, and kidney dysfunctions (El-Sheikh et al., 2012; Tulubas et al., 2015; Nagai et al., 2018). DXR exerts its pharmacological anticancer actions by targeting and intercalating DNA of rapidly dividing tumor cells, causing cell-cycle blockage in the G2 phase (Attia and Bakheet 2013). The exact molecular mechanisms of DXR-induced toxicity are not fully described; however, the most acceptable theory attributed to its toxicity is the initiation of oxidative stress (Korga et al., 2012). Reactive oxygen species (ROS) cause oncogenic mutations through direct interaction with DNA (Matsumura et al., 2018). EL-Sheikh and others investigated the mechanisms underlying DXR nephrotoxicity, focusing on the role of oxidative stress and apoptosis (El-Sheikh et al., 2012). Additionally, the ability of DXR to initiate the inflammatory cascade may be one of the factors causing organ toxicity (Park and Zheng 2012) and cell death (Zhang et al., 2009).

Several studies examined using antioxidants as probable protective adjuvant drugs to prevent DXR toxicity (Injac et al., 2009; Ayla et al., 2011; El-Moselhy and El-Sheikh, 2014; Liu et al., 2016; Alanazi et al., 2020; Sergazy et al., 2020). Carvedilol (Card) is a non-selective β-blocker with antioxidant and anti-inflammatory activities (Calò, Semplicini, and Davis 2005). Numerous studies proposed the ROS-scavenging activity of Card, and its use leads to reduction of oxidative stress (Nakamura et al., 2002; Spallarossa et al., 2004; Dandona et al., 2007; El-Shitany and El-Desoky, 2016).

Resveratrol (Resv) is a natural polyphenol and an active antioxidant compound. Resv exhibited anticancer activity in breast cancer, skin cancer, and liver cancer (Amiri et al., 2013; Junco et al., 2013; Chottanapund et al., 2014). Recently, it has been shown that Resv has a beneficial activity in improving cell survival and reducing the production of ROS (Monahan, et al., 2021). Moreover, using of Resv alleviated the toxicity of DXR through its anti-inflammatory and antioxidant activities (Alanazi, et al., 2020). Nano formulation is considered as a promising strategy that may improve the product’s dissolution and enhance its bioavailability (Isailović et al., 2013; Zu et al., 2018).

Hence, in this study, we were investigating the efficacy of nano-resveratrol (N-Resv) alone or in combination with Card, focusing on its autophagy regulatory mechanism. Cells use autophagy as a process to remove impaired proteins and organelles (Hale et al., 2013). This process is selectively switched in response to numerous types of cellular stresses (Fulda et al., 2010). The Beclin-1 gene plays an important role in activating autophagy *via* controlling the nucleation of autophagic vesicles (Kang et al., 2011; Wang et al., 2012). It was recorded that beclin-1 is activated in the autophagy process and induces the formation of resistance machinery in chemotherapy. Selectively downregulating beclin-1 gene expression can reduce the multidrug resistance and increase the sensitivity of cancer cells to DXR (Daniel et al., 2006). The mammalian target of rapamycin (mTOR) is a vital mediator of DXR-induced apoptosis and consequent autophagy dysregulation (Johnson et al., 2017). Autophagy and the mTOR are assumed to negatively regulate each other, as they have opposing molecular pathways (Fantus, et al., 2016). Cancer and metabolic dysfunction can be induced by deregulated mTOR signaling (Popova and Jücker, 2021).

It is well known that, due to the role of the kidney in the excretion of exogenous compounds, it is often involved in the adverse effect and toxicity induced by these agents. Drug-induced nephrotoxicity is triggered by various mechanisms including glomerular hemodynamics, inflammation, and disruption of functional protein expression levels (Yu, et al., 2022). Podocin is a critical component of the slit diaphragm in the glomerular filtration barrier (Serrano-Perez et al., 2018). Endoglin is a disulfide-linked, homodimeric transmembrane glycoprotein. It is an important co-receptor for the transforming growth factor-β (TGF-β) family and has an essential role in angiogenesis (Rossi, Bernabeu, and Smadja 2019). Moreover, it may affect tumor behavior by many sequence processes depending on the cell context. In some situations, endoglin promotes tumor development and progression, while in other situations it has a role in tumor suppression (Muñoz et al., 2021). Kidney injury molecule-1 (KIM-1), a type-1 cell surface glycoprotein, is expressed at a low level in the kidney, but it is highly overexpressed in injured renal tubule cells including human renal cell carcinoma, and it is considered an early marker for kidney cancer (Scelo, et al., 2018).

The purpose of this study was to investigate the effectiveness of using N-Resv alone or in combination with Card to limit DXR-induced renal toxicity *via* modulating the expression of endoglin, beclin-1, podocin, and mTOR.

## 2 Materials and Methods

### 2.1 Chemicals

Resv and Card raw powders were obtained from Sigma Chemical Co. (St. Louis, MO, USA), DXR was purchased from a local pharmacy in Riyadh (KSA), and the marketed N-Resv was purchased from Lipolife (Drakes Lane Industrial Estate, Drakes Lane, (UNited Kingdom), which was characterized as (Resv encapsulated in liposomes with particle size = 200 nm) by the manufacturer company.

### 2.2 Animals and Treatments

Forty-two adult male Wister rats weighing from 150 to 180 g were used in this study. The rats were obtained from the Animals Care Centre at the College of Pharmacy, King Saud University, Riyadh, Saudi Arabia. Animals were allowed to adapt to the laboratory condition for 1 week before commencing any experiments. They were kept at a temperature (25°C ± 2) with a 12-h light and dark cycle and provided with a balance of food and water. The experimental protocol was conducted according to the Institutional Animal Care and Use Committee at King Saud University (KSU-SE-18-31).

Rats were randomly divided into seven groups/six rats each. **Group 1** comprises the control group; rats were administered orally with the equivalent volume of 1% carboxymethylcellulose (CMC) as the vehicle of the drugs for 6 weeks; **Group 2** comprises the DXR-injected group; in the first week, rats were treated orally with 1% (CMC), then they received DXR (2 mg/kg, i.p.) twice weekly over 5 weeks to produce a total cumulative dose of 20 mg/kg (Tatlidede et al., 2009). **Group 3** comprises the Card-treated group; rats were pretreated with Card (30 mg/kg/day, p.o.) dissolved in 1% CMC for 6 weeks (Arozal et al., 2010). **Group 4** comprises the Resv-treated group; rats were pretreated with Res (20 mg/kg/day, p.o.) dissolved in 1% CMC for 6 weeks (Shoukry et al., 2017). **Group 5** comprises the N-Resv-treated group; rats were pretreated with N-Resv (20 mg/kg/day, p.o.) for 6 weeks. **Group 6** comprises the combination of Resv and Card-treated group; rats were pretreated with Card concomitantly with Resv for 6 weeks. **Group 7** comprises the combination of N-Resv- and Card-treated group; rats were pretreated with Card concomitantly with N-Res for 6 weeks.

In all treated groups, DXR was administered in the second week (2 mg/kg, i.p.) twice weekly for 5 weeks, along with antioxidants as mentioned above.

After completing all treatments, rats were subjected to a gradually increasing concentration of carbon dioxide (Thomas, Flecknell, and Golledge 2012) and sacrificed by decapitation. Blood samples were collected; serum was separated by centrifugation at 3,000 rpm at 4°C for 20 min. Kidneys were removed, and one part was placed in 10% formalin for histopathological studies. Other parts were immediately frozen in liquid nitrogen and stored at -80°C for Western blot analysis. The rest of the kidney tissue was homogenized (20% w/v) in phosphate-buffered saline and centrifuged at 10,000 rpm at 4°C for 20 min, then the supernatants were collected and stored at −80°C with the obtained serum for biochemical and molecular analyses.

### 2.3 Biochemical Serum Analysis

#### 2.3.1 Determination of Kidney Function Parameters

Estimations of serum creatinine and urea were performed using kits obtained from Randox Company.

#### 2.3.2 Determination of renal oxidant/antioxidant biomarkers

The renal lipid peroxidation (MDA) level was evaluated as previously described (Mihara and Uchiyama 1978). Briefly, the kidney tissue homogenate (20% w/v) in phosphate-buffered saline was mixed with SDS, TBA, and acetate buffer and then placed in boiling water (95 C) bath for 60 min. After cooling, n-butanol was added to the mixture, then centrifuged, and the absorbance of the organic layer was measured at 532 nm. Renal GSH was estimated according to the Ellman method, and this procedure is based on the reaction between GSH and 5,50-dithio-bis(2-nitrobenzoic acid), and the absorbance of the yellow-colored product is measured at 412 nm (Ellman 1959).

### 2.4 Western Blotting Analysis

The expression levels of beclin-1 and mTOR proteins in kidney tissue were evaluated using Western blotting analysis (Mahmood and Yang 2012). Briefly, kidney tissue lysates were separated by SDS-PAGE and blotted into the PVDF membrane. The nonspecific binding was blocked with 5% non-fat milk for 2 h, then the primary antibody was incubated overnight at 4°C. Finally, the corresponding secondary antibody was used for signal detection by chemiluminescence. The immunoreactivity was visualized by ImageQuant LAS 4000 and quantified by ImageJ software.

### 2.5 Real-Time PCR Analysis of KIM-1, Podocin and Endoglin

Gene expression was detected using real-time PCR according to specific forward and reverse primers, as follows: KIM-1 forward primer: 5′-TAT​TTG​GGG​GAA​CAG​GTT​GC-3′; reverse primer: 5′-CAA​GTC​ACT​CTG​GTT​AGC​CGT​G-3′; podocin forward primer: 5′-TGG​AAG​CTG​AGG​CAC​AAA​GA-3′; reverse primer: 5′-AGA​ATC​TCA​GCC​GCC​ATC​CT-3′; and endoglin forward primer: 5′-CTC​CCG​GGT​GGA​CAG​C-3′; reverse primer: 5′-AGG​CTC​CAG​GCT​GGG​T-3′. Firstly, total RNA was extracted from kidney samples using the SV Total RNA Isolation System (Promega, Madison, WI), then the extracted RNA was reverse transcribed into cDNA and amplified by PCR using the RT-PCR kit (Stratagene, La Jolla, CA, USA). The cycling parameters were optimized for each gene, and the PCR products were loaded into 1.5%–3.5% agarose/ethidium bromide gel. The changes in gene expression were determined as a fold change relative to control.

### 2.6 DNA Fragmentation

DNA fragmentation was quantified by measuring the cytosolic nucleosomes using a Cell Death ELISA kit (Roche Diagnostics GmbH, Indianapolis, United States).

### 2.7 Histopathological Analysis

Kidney specimens were excised and stored in 10% formalin. Sections were cut into 4-µm thicknesses and used for histopathological examination after hematoxylin and eosin (H&E) staining.

### 2.8 Statistical analysis

The statistical analysis was performed using GraphPad Prism (GraphPad Software, San Diego, CA, USA). All statistical comparisons were performed by one-way analysis of variance test followed by Tukey’s test *post hoc* analysis. Results were expressed as mean ± SEM, and a *p*-value ≤ 0.05 was considered significant.

## 3 Results

### 3.1 N-Resv and Card Improved the Renal Function After DXR Induced Nephrotoxicity

**FIGURE 1:**
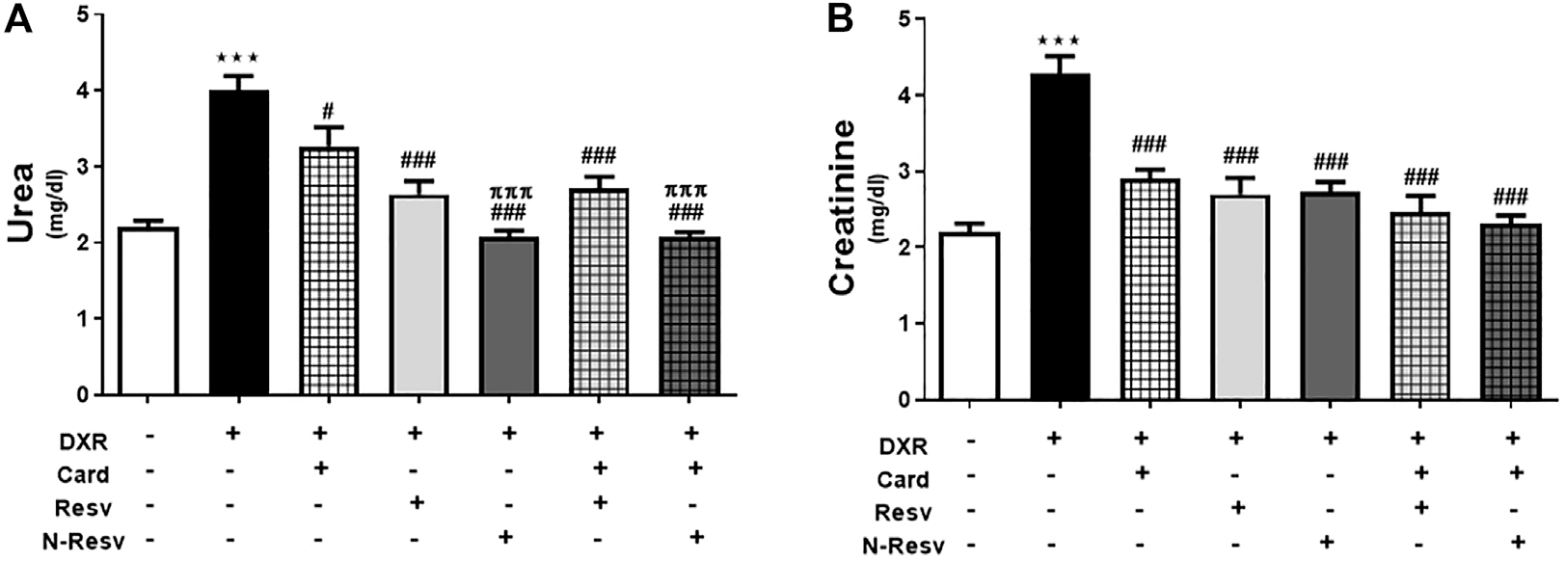
Card and N-Resv ameliorate urea **(A)** and creatinine **(B)** after DXR-induced nephrotoxicity in rats. Data are expressed as mean ± SEM (*n* = 6). ^***^ *p*  ≤ 0.001 versus Control rats, ^###^ *p*  ≤  0.001, ^#^ *p*  ≤ 0.05 versus DXR-intoxicated rats, and ^πππ^p  ≤ 0.001 versus Card-treated rats.

Renal function tests of DXR-treated and all treated groups are presented in Figure 1, where creatinine and urea were measured in serum. DXR administration significantly increased the urea (*p* ≤ 0.001, Figure 1A) and creatinine levels (*p* ≤ 0.001 Figure 1B). Groups treated with the antioxidants singly or simultaneously showed a significant decrease in urea and creatinine relative to the DOX-intoxicated group. N-Resv alone or with Card showed a remarkable reduction in urea (*p* ≤ 0.001), but not creatinine, compared to Card.

### 3.2 N-Resv and Card Reversed the Oxidative Stress After DXR Induced Nephrotoxicity

**FIGURE 2:**
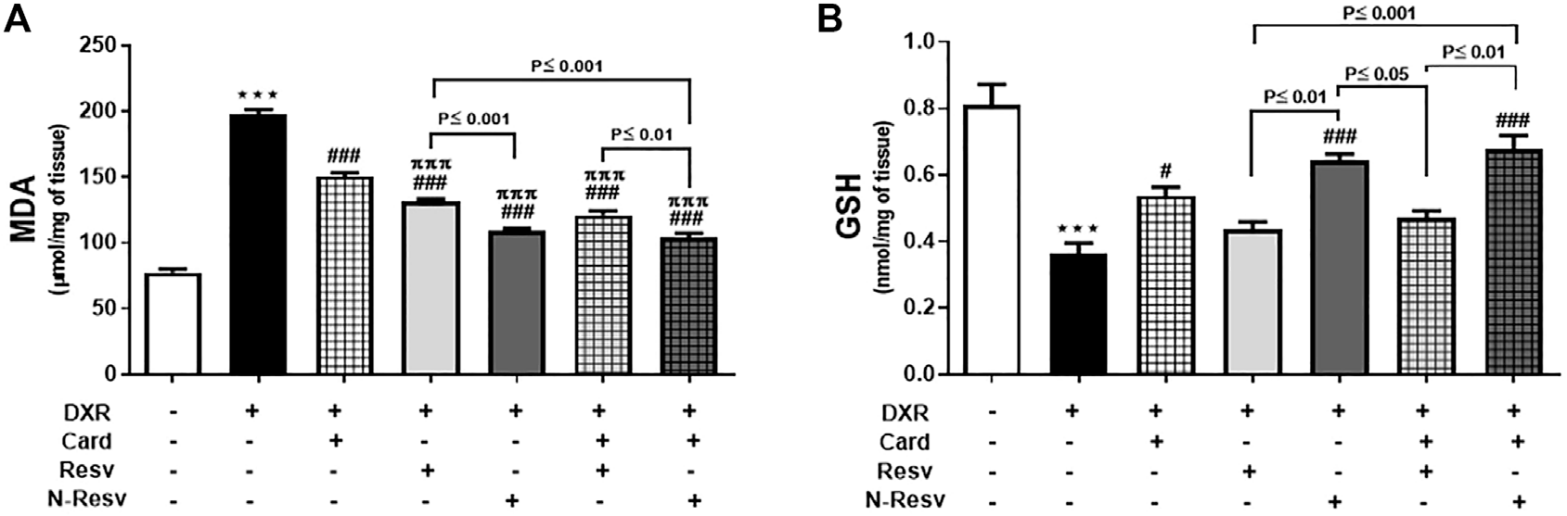
Card and N-Resv decrease renal MDA **(A)** and increase GSH **(B)** levels after DXR-induced nephrotoxicity in rats. Data are expressed as mean ± SEM (*n* = 6). ^***^ *p*  ≤ 0.001 versus Control rats, ^###^ *p*  ≤ 0.01, ^#^ *p*  ≤ 0.05 versus DXR intoxicated rats, and ^πππ^p  ≤ 0.001 versus Card treated rats.

Oxidative stress markers were measured to evaluate the antioxidant properties of Card, Resv, N-Resv, and their combinations against DXR nephrotoxicity. As expected, DXR significantly elevated the MDA (*p* ≤ 0.001, Figure 2A) and reduced the GSH (*p* ≤ 0.001, Figure 2B) levels in the renal tissues of the intoxicated rats compared to those in the control rats. However, the use of these antioxidants alone or simultaneously markedly lowered the MDA in which N-Resv and Card + N-Resv showed potent effects and returned MDA close to the control levels. On the other hand, GSH was significantly increased with Card, N-Resv, and Card + N-Resv, but Resv was not effective enough to restore the GSH normal level.

### 3.3 N-Resv and Card Mitigated the DXR Renal Toxicity *via* Modulating Beclin-1 and mTOR Protein Expression

**FIGURE 3:**
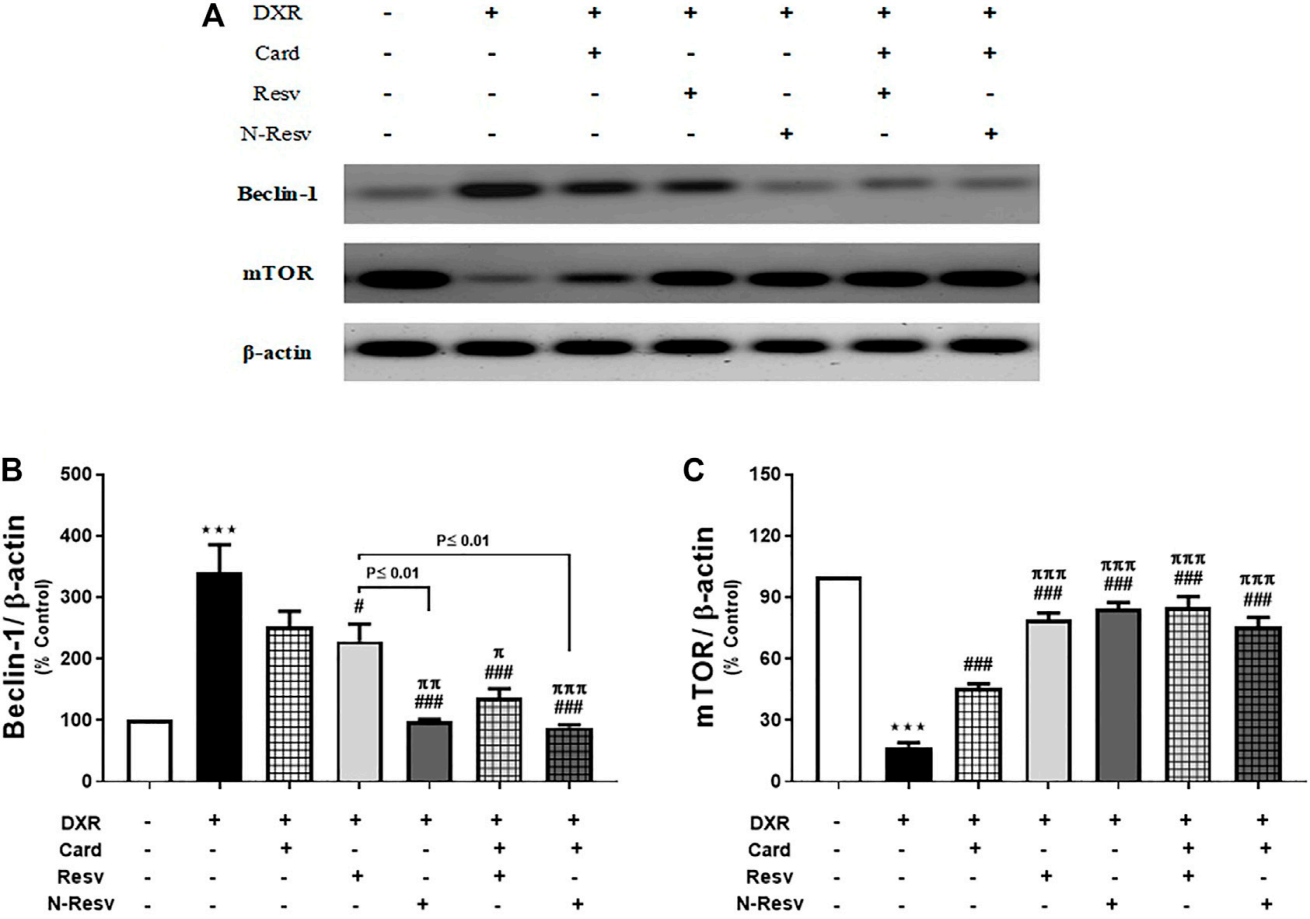
Effect of Card and/or Resv and N-Resv on the protein expression of renal beclin-1 and mTOR after DXR intoxication: Representative Western blot of fractionated samples **(A)**. Quantitative analysis of the protein expression of renal beclin-1 **(B)** and mTOR **(C)**. Data are expressed as mean ± SEM (*n* = 6). ^***^ *p* ≤ 0.001 versus Control rats, ^###^ *p* ≤ 0.001, ^#^ *p* ≤ 0.05 versus DXR-intoxicated rats, ^πππ^p ≤ 0.001, ^ππ^p ≤ 0.01, and ^π^p ≤ 0.05 versus Card-treated rats.

DXR administration altered the renal expression of beclin-1 and mTOR proteins (Figure 3), in which beclin-1 was significantly upregulated (*p* ≤ 0.001, Figure 3B), while mTOR was significantly downregulated (*p* ≤ 0.001, Figure 3C) in comparison to the control rats. Card was weak to reduce beclin-1 (*p* ≥ 0.05), but strong to increase mTOR (*p* ≤ 0.001). Resv and N-Resv with and without Card ameliorated these alterations in such proteins significantly and made them close to control expression. In addition, N-Resv had a superior action on beclin-1 compared to its regular formulation.

### 3.4 N-Resv and Card Alleviated the DXR Renal Toxicity *via* Modulating Podocin, KIM-1 and Endoglin Genes

**FIGURE 4:**
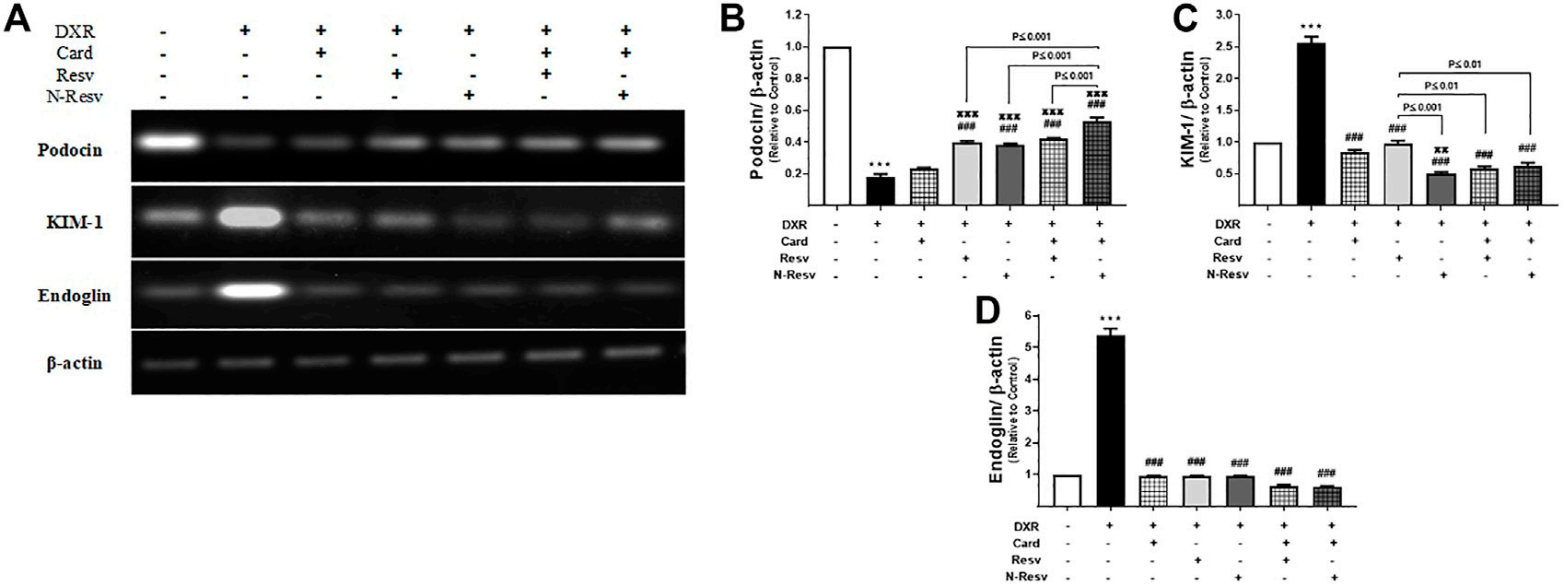
The effect of DXR on podocin, KIM-1, and endoglin mRNA expression in control and different treated groups: representative RT-PCR **(A)**. Quantitative analysis of podocin **(B)**, KIM-1 **(C),** and endoglin **(D)** mRNA expression. Data are expressed as mean ± SEM (*n* = 6). ^***^ *p* ≤ 0.001 versus Control rats, ^###^ *p* ≤ 0.001 versus DXR-intoxicated rats, ^πππ^p ≤ 0.001, and ^ππ^p ≤ 0.01 versus Card-treated rats.

Figure 4A shows the expression of renal podocin, KIM-1, and endoglin measured by RT-PCR. The intake of DXR significantly caused a marked reduction in mRNA expression of podocin (*p* ≤ 0.001, Figure 4B) and increased gene expression of KIM-1 (*p* ≤ 0.001, Figure 4C) and endoglin (*p* ≤ 0.001, Figure 4D). All tested antioxidants significantly ameliorated DXR effects on these genes at a variable level.

### 3.5 N-Resv and Card Prevented Renal DNA Damage in Response to DXR

**FIGURE 5:**
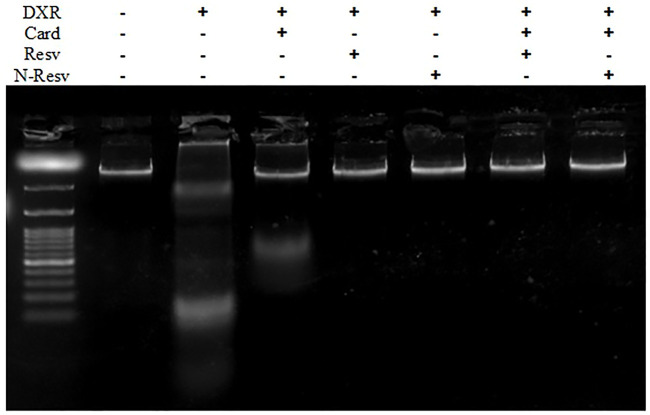
DNA fragmentation analysis of the renal tissue in control, DXR-intoxicated, and DXR-treated rats.

The effects of Card, Resv, and N-Resv against DXR-induced renal cell damage were assessed by DNA fragmentation analysis (Figure 5). DXR-intoxicated rats showed an apparent fragmentation of genomic DNA indicated by the presence of a smeared band in agarose gel (Lane 2). Likewise, Card on its own was not effective enough to protect the DNA integrity; however, it preserved some of the DNA as appeared in the top of lane 3. Other treatments prevented the damaging effect of DXR on DNA and kept it intact.

### 3.6 N-Resv and Card Prevented Histopathological Changes in Renal Tissue After DXR Overdose

**FIGURE 6:**
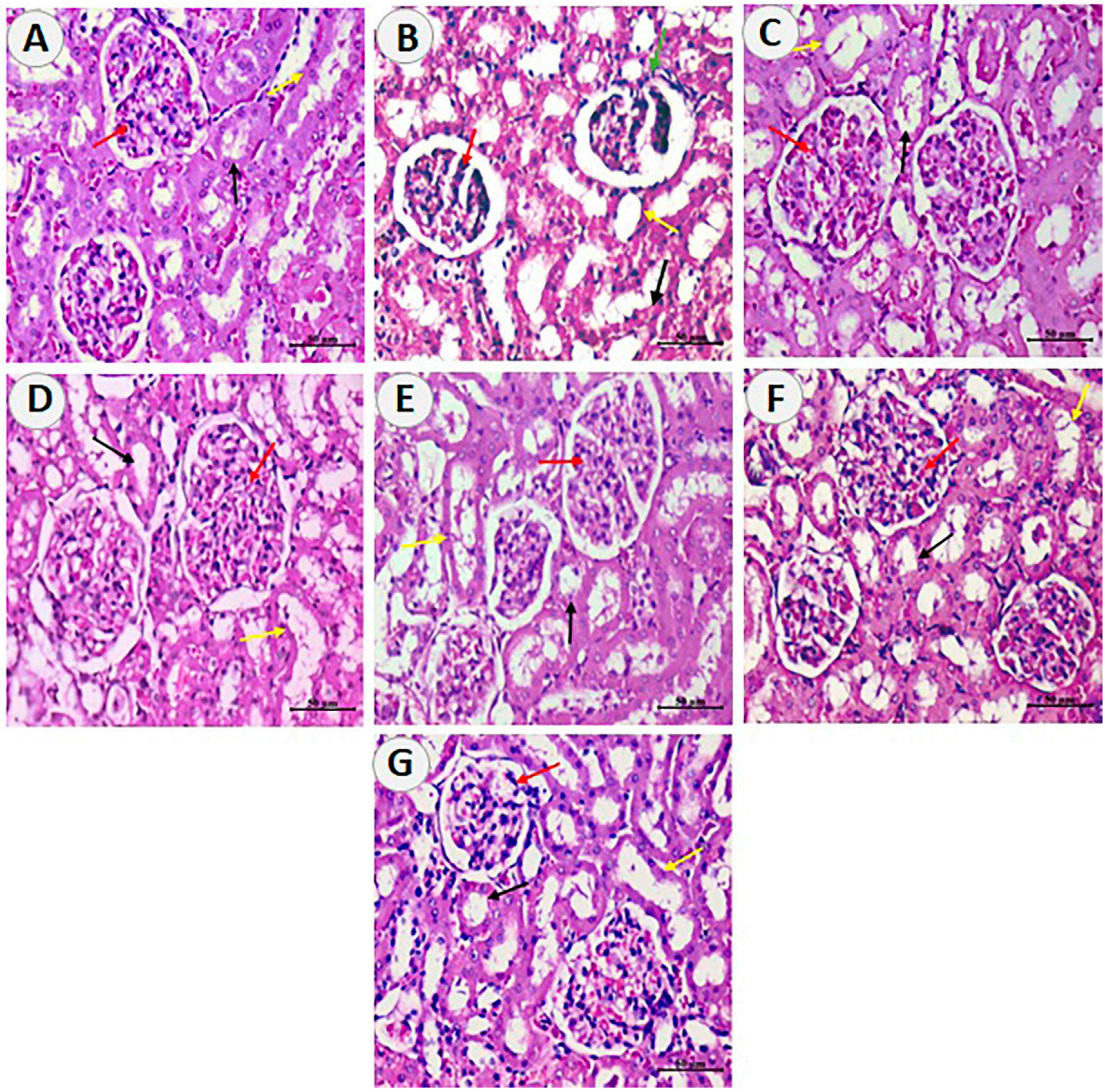
Photomicrographs of sections from renal tissue of **(A)** control rats showing the normal renal cortex, corpuscle and glomerulus, and normal pattern of proximal and distal convoluted tubules. **(B)** DXR-intoxicated rats showing few destructed glomeruli corpuscles (green arrows) and cellular hyperplasia of epithelial cells lining the partial layer of Bowman’s capsule, and degenerated epithelial lining of proximal and distal convoluted tubules. **(C)** Card-treated rats showing mild improvement in renal cortex corpuscle and glomerulus and mildly dilated proximal and distal convoluted tubules. **(D)** Resv-treated rats and **(E)** N-Resv-treated rats showing renal corpuscle with mild cellularity (hyperplasia of epithelial cells lining the partial layer of Bowman’s capsule), and mildly dilated proximal and distal convoluted tubules. **(F)** Card + Resv-treated rats showing mild cellular corpuscle and almost normal proximal and distal convoluted tubules. **(G)** Card + N-Revs-treated rats showing normal corpuscle with glomerulus and normal proximal(black arrow) and distal (yellow arrow) tubules. The Bowman’s capsule and its structure are denoted by red arrows, proximal tubules by black arrows, and distal tubules by yellow arrows.

The histopathological evaluation was used to assess the nephrotoxic effects of DXR and protective effects of Card, Resv, and N-Resv. While the control rats (Figure 6A) showed normal renal architecture, examining renal sections after DXR revealed regions of degenerated glomeruli corpuscles, hyperplasia, and destructed convoluted tubules (Figure 6B). Treatment with Card showed mild glomerular degeneration and tubule dilatation (Figure 6C), whereas Resv-treated rats (Figure 6D) and N-Resv-treated rats (Figure 6E) revealed mild hyperplasia and tubule dilatation. Treatment with Card + Resv (Figure 6F) and Card + N-Resv (Figure 6G) reversed these changes and showed almost normal corpuscles and tubules.

## 4 Discussion

DXR is an anthracycline antibiotic that interacts with cellular DNA and exhibits an effective antineoplastic activity against several types of cancer (Attia and Bakheet 2013). Despite its powerful effect in cancer treatment, the associated risk of cardiotoxicity and nephrotoxicity limits its use (Tulubas et al., 2015; Nagai et al., 2018). Therefore, new liposomal formulas of DXR were designed to overcome these toxicities. However, nephrotoxicity was reported among some patients who received pegylated liposomal doxorubicin (PLD) for a long time (Kwa et al., 2012). As reported previously, free radicals generated by DXR could induce lipid peroxidation of glomerulus leading to abnormal renal function and subsequent metabolic disorders (Pérez-Arnaiz et al., 2014; Heart et al., 2016). Numerous phytochemicals were found to defeat cancer cells without disturbing normal cells, exert chemoprophylaxis activities, support cancer cells’ sensitivity to DXR (An et al., 2017), and improve chemotherapy-associated side effects (Choudhari et al., 2020). Resv has attracted growing interest due to its diverse, health benefits, including antioxidant activity and nephroprotection against several toxicants (Novelle et al., 2015; Rajagopal et al., 2018), specifically if used in its nanoformulation (Barbosa et al., 2019; Sharifi-Rad et al., 2021). More recently, Sharifi-Rad and others emphasized the distinctive role of N-Resv in cancer treatment as it contributes to autophagy induction in cancer cells and inhibits angiogenesis and metastasis, thus adding to other chemotherapy actions and minimizing their side effects (Sharifi-Rad et al., 2021). Besides phytochemicals, some medications like Card (non-selective β-blocker) present antioxidant, free radical scavenger, lipid peroxidation inhibitor, and calcium antagonist properties (Singh, Chander, and Chopra 2004; Smyth and Daly 2008). Furthermore, Card showed beneficial outcomes for breast cancer as it slowed cancer invasion and growth *in vivo* and reduced cancer-associated mortality in humans (Gillis et al., 2021).

In the current study, the effects of Card, Resv, and N-Resv either alone or in combination on DXR-induced nephrotoxicity were investigated in the light of biochemical parameters and histopathological investigations. DXR clinical use is restricted due to its damaging effect on both the kidney and heart. Previous examinations proposed that oxidative stress is linked with antioxidant defense status alteration, triggering the cascade of reactions accountable for DXR-induced cardiotoxicity and nephrotoxicity (Nakamura et al., 2000; Kumar et al., 2001). DXR generates free radical either by NADPH reductases, which produces superoxide radicals in the occurrence of oxygen, or by reacting with iron and reducing O_2_ to H_2_O_2_ (Abo-Salem 2012). Disturbance in the rate of ROS production and the defense system of the cell results in cell damage, and this imbalance plays a vital role in different disease progressions (Quiles et al., 2002; Injac et al., 2009). GSH reduced level is correlated with the drop of the cells’ defense against ROS-prompted damage, causing necrotic cell death (Mahmoud and Al Dera 2015). Deman and others revealed that depletion in GSH concentration in the cortex of the kidney enforces the concept of free radical generation leading to DXR nephrotoxicity (Deman et al., 2001). Moreover, it has been shown that an intraperitoneal dose of DXR for 10 days increased the renal MDA level and reduced the GSH level (Yagmurca et al., 2004; Refaie et al., 2016). The results of those studies matched with the current findings which revealed a decrease in renal GSH content and an increase in MDA activity in DXR-injected rats. Treating animals with N-Resv combined with Card suppressed the nephrocellular oxidative stress activity induced by DXR and restored the GSH level. This nephroprotective activity results from the antioxidant effect of N-Resv and Card which was reflected on the restored levels of urea and creatinine in serum.

Cellular autophagy is a process to remove impaired proteins and organelles from intracellular space (Hale et al., 2013). This process is selectively switched on to respond to numerous cellular stresses (Fulda et al., 2010). DXR induces such stress exhibiting nephrotoxicity *via* the initiation of apoptosis with concurrent autophagy reduction (Yi et al., 2017). Moreover, it was reported that DXR increased renal beclin-1 protein expression while decreasing mTOR expression (An et al., 2017; KozaK et al., 2018; Zilinyi et al., 2018). Herein, injection of DXR induced the upregulation of beclin-1 with concurrent depletion in mTOR protein expression levels. Several studies have reported the inhibitory effect of Resv and the machinery of its effect on tumor cells (Mahyar-Roemer et al., 2001; Tan et al., 2016; Buhrmann et al., 2017; Qin et al., 2020). Co-administration of Resv during DXR treatment is satisfactory to normalize nephrotoxicity markers (Oktem et al., 2012). In addition, it was demonstrated that Resv induces autophagy by directly inhibiting the mTOR-ULK1 pathway or indirectly *via* acting on SIRT1 or inhibiting mTORC1 activity through stimulating AMPK (D. Park et al., 2016; Y. Tian et al., 2019). Beclin-1 is involved in the initial step of Resv-mediated formation of the autophagosome; Resv could reduce the extent of autophagy marker beclin-1 and induce mTOR phosphorylation at the serine 2481 amino acid position (Gurusamy et al., 2010). Interestingly in the current study, treatment with N-Resv with or without Card decreased beclin-1 protein expression compared to other groups of antioxidants. However, N-Resv alone or co-administered with Card did not show significant differences compared with other antioxidant-treated groups in increasing mTOR expression in rat kidneys. Therefore, N-Resv could lower the autophagy process in renal tissue at the early stages. This finding is consistent with previous data obtained by Gurusamy’s group (Gurusamy et al., 2010), where the high dose of Resv could attenuate autophagy in cardiac tissue. Thus, this Resv behavior could be used to enhance sensitivity to DXR in some types of cancer (Chen et al., 2018).

To determine the effect of DXR in renal tissue, the mRNA level of several markers for nephrocellular injury was used in the current studies, including podocin, KIM-1, and endoglin. Podocin is an essential protein expressed in podocytes that cover the glomerular filtration barrier, and it belongs to the stomatin and prohibitin homology domain protein family (Huber et al., 2006). According to literature, reduction in podocin level in the kidney is associated with nephropathies (Kandasamy et al., 2014). Based on previous data, KIM-1 is an inducible transmembrane protein and it is an important marker for indicating and predicting kidney injury (L. Tian et al., 2017). The last marker used in this study is endoglin, a glycoprotein that interacts with TGF-β to induce fibrosis in several organs such as the liver and heart (Gerrits et al., 2020). In kidney biopsy obtained from diabetic nephropathy patients, the endoglin level significantly increases compared to the control (Gerrits et al., 2020). Thus, DXR is one of the antineoplastic agents associated with renal tissue injury. The current investigation presents that DXR significantly caused a reduction in mRNA expression of podocin and increased the gene expression of KIM-1 and endoglin compared to those in the control group. When the animals were treated with the antioxidants under study, podocin, KIM-1, and endoglin mRNA expression levels were restored to their normal levels. N-Resv combined with Card expressed a stronger effect of neutralizing the podocin level than other treated groups. Moreover, treating animals with Resv or N-Resv in the presence or absence of Card exhibited a protective activity against DNA fragmentation and renal cell injuries. According to Spallarossa’s research, Card pretreatment is associated with a high rate of DNA preservation in DXR-induced cardiotoxicity *in vitro* (Spallarossa et al., 2004).

In the current study, the nephroprotective mechanisms of Resv and N-Resv in the presence or absence of Card were reflected on the histopathological observations of the rat kidney injured by DXR. The antioxidants preserved renal glomerulus capsules with no sign of epithelial cell hyperplasia and normalizing proximal and distal tubules. These effects were more observed in rats treated with N-Resv combined with Card. DXR acute toxicity could be reversible with adequate treatment; however, the life expectancy of patients possessing DXR-induced renal failure is currently unclear. The underlying mechanism appears to be multifactorial; however, enhanced oxidative stress is one of the major contributors (Chatterjee et al., 2010). Indeed, cells fail to cope with an enhanced amount of reactive oxygen and nitrogen species, and enhanced oxidative stress leads to DNA and protein damage and mitochondrial dysfunction (Cappetta et al., 2017).

To sum up, the studied antioxidant co-administration, especially Resv or N-Resv combined with Card, successfully ameliorated all the preceding protein and mRNA expression parameters and DNA degradation. Furthermore, using Resv or N-Resv with Card potentiated the nephroprotective activity compared with when each agent is used alone. The synergistic activity may result from the combination of different kidney-protective mechanisms produced by Resv/N-Resv and Card. Some of these mechanisms are the antioxidant and anti-inflammatory activities of Resv/N-Resv and Card, modulating angiogenesis by Resv/N-Resv and preserving mitochondrial functions by Card (Rodrigues et al., 2010; Gowd et al., 2020). Interestingly, the result shows that N-Resv is associated with relatively more autophagy inhibition and potent antioxidant effects than Resv. This observation could result from the enhanced bioavailability and plasma membrane transportation of the nanoencapsulation Resv (Zu et al., 2018).

The current study is limited by the lack of assessment of the possible changes in the anticancer activity of DXR in the presence of Card and N-Resv. The assessment of how Resv or card can alter the DXR anticancer activity was also not explored in previously reported studies but should be in the future. Nowadays, phytochemicals take the scientists’ attention with their potentials to treat diseases and the side effects of medication use. Therefore, the presence of different formulations to enhance the bioavailability of phytochemicals is important. However, studying the effect of changing their bioavailability at the pharmacological and toxicological levels is critical in order to maximize the benefits while avoiding such toxicity.

## Data Availability

The original contributions presented in the study are included in the article/supplementary material, further inquiries can be directed to the corresponding author.
